# *Rickettsia parkeri* and *Rickettsia montanensis*, Kentucky and Tennessee, USA

**DOI:** 10.3201/eid2010.140175

**Published:** 2014-10

**Authors:** Benedict B. Pagac, Melissa K. Miller, Meagan C. Mazzei, David H. Nielsen, Ju Jiang, Allen L. Richards

**Affiliations:** US Army Public Health Command Region North, Fort George G. Meade, Maryland, USA (B.B. Pagac, M.K. Miller, M.C. Mazzei, D.H. Nielsen);; Naval Medical Research Center, Silver Spring, Maryland, USA (J. Jiang, A.L. Richards)

**Keywords:** Amblyomma maculatum, Dermacentor variabilis, ticks, Rickettsia parkeri, Rickettsia montanensis, rickettsia, bacteria, military training sites, Kentucky, Tennessee

## Abstract

We found that 14.3% (15/105) of *Amblyomma maculatum* and 3.3% (10/299) of *Dermacentor variabilis* ticks collected at 3 high-use military training sites in west-central Kentucky and northern Tennessee, USA, were infected with *Rickettsia parkeri* and *Rickettsia montanensis*, respectively. These findings warrant regional increased public health awareness for rickettsial pathogens and disease.

The Gulf Coast tick (*Amblyomma maculatum*) has become well-established in states outside its historically described coastal range, most recently in North Carolina and Virginia (*1**,**2**)*. This tick has been sporadically reported in other states, including Tennessee and Kentucky (*3**,**4*). *A. maculatum* ticks are the recognized vector of *Rickettsia parkeri*, a spotted fever group (SFG) bacterium that is pathogenic to humans and has caused illness in ≥32 patients (*5**–**7*; C. Paddock, unpub. data).

*R. parkeri*–infected *A. maculatum* ticks from Kentucky were among specimens submitted to the human tick–testing program of the US Army during 2000–2009 (*4*), which increased concern of a potential health threat to military personnel using field training areas. To assess the threat of human exposure to *R. parkeri* and other potential rickettsial pathogens, we conducted a tick survey at 3 high-use military training sites in west-central Kentucky and northern Tennessee, USA.

## The Study

Questing ticks were collected during July 16–20, 2012, by using cloth drags, flags, and CO_2_-baited traps, and by removing ticks from collectors (Table 1). Sites of collection were Fort Campbell (Christian County, Kentucky, and Montgomery County, Tennessee), Fort Knox (Bullitt, Hardin, and Meade Counties, Kentucky), and Wendell H. Ford Regional Training Center (WHFRTC; Muhlenberg County, Kentucky).

**Table 1:** Quantitative PCR results for rickettsia in *Amblyomma maculatum* and *Dermacentor variabilis* ticks, Kentucky and Tennessee, USA, 2012

Location, tick species	No.	No. (%) positive for *Rickettsia parkeri*	No. (%) positive for R. *montanenesis*
Fort Knox, Kentucky			
* A. maculatum*	3	0	0
* D. variabilis*	44	0	2 (5)
Fort Campbell, Kentucky and Tennessee			
* A. maculatum*	66	10 (15)	0
* D. variabilis*	148	0	6 (4)
Wendell Ford Regional Training Center, Kentucky			
* A. maculatum*	36	5 (14)	0
* D. variabilis*	107	0	2 (2)
Total			
* A. maculatum*	105	15 (14)	0
* D. variablilis*	299	0	10 (3)

Multiple 2-person teams collected ticks during 15-minute periods; an average of 19 person-hours was spent sampling at each site. Target tick species were *A. maculatum* and *Dermacentor variabilis*, although *A. americanum* ticks were also collected. Human encounter rates (calculated by using all collection methods except CO_2_-baited traps) for adult *A. maculatum* and *D. variabilis* ticks were ≈2 ticks/hour and 5 ticks/hour, respectively. No immature stages of these species were encountered. Field sites sampled were dominated by sericea (*Lespedeza cuneata*) and fescue (*Festuca pratensis*). Some adjacent areas had switchgrass (*Panicum virgatum*) and Indiangrass (*Sorghastrum nutans*). *A. maculatum* ticks appeared tolerant of exposed, unshaded sites and were often collected in the middle of these fields.

Ticks were identified by using the key of Keirans and Litwak (*8*). Specimens were individually placed in microcentrifuge tubes containing 500 μL of tissue lysis buffer (QIAGEN, Valencia, CA, USA) and 20 μL of proteinase K (QIAGEN), bisected with a sterile blade, and incubated at 56°C for ≥1 h. Nucleic acid was extracted by using the DNeasy Blood and Tissue Kit (QIAGEN).

Initial quantitative real-time PCRs (qPCRs) were performed by using the *Rickettsia*-specific Rick17b assay specific for the 17-kD antigen gene (*4*) and the LightCycler TaqMan Master (Roche Applied Sciences, Indianapolis, IN, USA) ready-to-use hot start reaction mixture in the LightCycler 2.0 instrument (Roche Applied Sciences). Final reactions contained 5 μL of template and 15 μL of master mixture. Master mixture contained 0.5 µmol/L primers, 0.4 μmol/L probe, LightCycler TaqMan Reaction Mixture (Roche Applied Sciences), and water. All qPCRs were performed at 95°C for 10 min and for 45 cycles at 95°C for 15 s and 60°C for 30 s.

Positive samples were further evaluated by using the SFG *Rickettsia*-specific conventional PCR with primer pair Rr190.70p and Rr190.602n, which is specific for the outer membrane protein A (*ompA*) gene of *Rickettsia* spp. and speciated by using *Pst*I restriction fragment length polymorphism analysis (*9*). Identities of 9 positive samples were confirmed by sequencing a fragment of *ompA* (1,651 bp) or *ompB* (1,540 bp) genes (Table 2) (*10*). All *A. maculatum* tick samples positive for *Rickettsia* spp. were also tested for *Candidatus* Rickettsia andeanae by using the Rande qPCR (*4*).

**Table 2:** Primers used for PCR, nested PCR, and sequencing for *Rickettsia parkeri* and *Rickettsia montanensis*, Kentucky and Tennessee, USA, 2012*

Gene, primer	Sequence (5′→3′)	Fragment, bp
*ompB*		
120-M59	CCGCAGGGTTGGTAACTGC	
ompB1570R	TCGCCGGTAATTRTAGCACT	PCR: 1,540
120–607F	AATATCGCTGACGGTCAAGGT	
120–807R	CCTTTTAGATTACCGCCTAA	
*ompA*		
190–3588F	AACAGTGAATGTAGGAGCAG	
RompA3182R	TTGCTGAGCGAAAYACTTACTYC	PCR: 3,202
190–5238R	ACTATTAAAGGCTAGGCTATT	Nested PCR: 1,651
RhoA4336F	AGTTCAGGAAACGACCGTA	
RompA4433R	TTTCCTGCAGTTACAGAATTTAAT	

*omp, outer membrane protein.

A total of 404 adult ticks (105 *A. maculatum* and 299 *D. variabilis*) were collected and tested. Of these ticks, 3 *A. maculatum* and 44 *D. variabilis* ticks were collected from Fort Knox, 66 *A. maculatum* and 148 *D. variabilis* ticks were collected from Fort Campbell, and 36 *A. maculatum* and 107 *D. variabilis* ticks were collected from WHFRTC. Twenty-five (6.2%) of 404 ticks were infected with an SFG *Rickettsia* species. *R. parkeri* was detected in 15 (14.3%) of the *A. maculatum* ticks.

The *ompA* sequences (GenBank accession no. KJ741849) of *A. maculatum* ticks collected from Fort Campbell (n = 2) and WHFRTC (n = 2) were identical to those of *R. parkeri* strain Portsmouth (GenBank accession no. CP003341) and *R. parkeri* Maculatum 20 (GenBank accession no. U83449). *R*. *montanensis* was detected in 10 (3.3%) of the *D. variabilis* ticks; isolates from 5 tick samples were sequenced. The *ompA* sequences (GenBank accession no. KJ741850) of *D. variabilis* ticks from Fort Knox (n = 1) and WHFRTC (n = 2) were 99.9% identical with *R*. *montanensis* str. OSU 85–930 (GenBank accession no. CP003340). The *ompB* sequences (GenBank accession no. KJ741851) of 2 *D*. *variabilis* ticks collected at Fort Campbell were 99.9% identical with those of *R*. *montanensis* str. OSU 85–930 (GenBank accession no. CP003340). No other *Rickettsia* spp., including *R. rickettsii*, were detected in any of the 404 ticks tested. The greatest percentage (15%) of *R. parkeri*–positive *A. maculatum* ticks were from Fort Campbell. *R. parkeri* was not detected in any of the *A. maculatum* ticks from Fort Knox.

## Conclusions

Given that *A. maculatum* ticks were collected at multiple sites during multiple years, and that these ticks have recently been collected in large numbers, this species is probably established in west-central Kentucky and northern Tennessee. To further elucidate its distribution, efforts should be made to collect immature stages of *A. maculatum* ticks from hosts, particularly birds, throughout both states.

The etiologic agent of Rocky Mountain spotted fever (RMSF), *R. rickettsii,* was not found in any of the ticks analyzed during this study, a finding that is consistent with findings of Fritzen et al. (*11*). However, during 2008–2012, a total of 15 human RMSF cases (5-year average rate of 0.1 cases/100,000 population) were reported to the Kentucky Department of Public Health (*12*). Likewise, for the same period, 1,695 cases of RMSF were reported to the Tennessee Department of Health (5-year average of 393 cases/100,000 population) (*13*). In addition, an *R. parkeri* human infection in Kentucky has been confirmed by PCR analysis of a tissue biopsy specimen from a patient (*5*). Thus, persons in west-central Kentucky and northern Tennessee may be more likely to become infected with a rickettsial agent other than *R. rickettsii.*

The tick encounter rates during this study suggest that persons entering appropriate habitats, especially for an extended period, are likely to encounter *D. variabilis* and *A. maculatum* ticks in west-central Kentucky and northern Tennessee during mid-summer. This study further suggests that although a person is ≈2.5 times more likely to encounter *D. variabilis* ticks than *A. maculatum* ticks, persons are ≈4.5 times more likely to encounter an *R. parkeri*–positive *A. maculatum* tick than a rickettsia-positive *D. variabilis* tick. These results are consistent with those of Stromdahl et al. (*14*).

Further evidence is needed to confirm if *R. montanensis* in *D. variabilis* ticks is of medical concern, but there has been 1 report of tick-borne *R. montanensis* infection associated with a nonfebrile episode in a person with a rash (*15*). Because of the lack of awareness regarding *R. montanensis* infection, it is plausible that a rash could be misdiagnosed and assumed to be a sign of a different illness. Even if an illness was recognized as a vectorborne disease, rickettsial serologic assays are not able to distinguish 1 species of SFG rickettsia from another (*14*). This finding indicates that serologic reactivity caused by exposure to *R. montanensis* could be attributed to the wrong SFG rickettsiae. Other epidemiologic studies are needed to elucidate how these findings may relate to regional rickettsial illness, but they still confirm that *A. maculatum* ticks infected with *R. parkeri* and *D. variabilis* ticks infected with *R. montanensis* warrant increased public health awareness in this region.
